# Matrix metalloproteinase activity stimulates N-cadherin shedding and the soluble N-cadherin ectodomain promotes classical microglial activation

**DOI:** 10.1186/s12974-017-0827-4

**Published:** 2017-03-17

**Authors:** Katherine Conant, Stefano Daniele, P. Lorenzo Bozzelli, Tsion Abdi, Amanda Edwards, Arek Szklarczyk, India Olchefske, David Ottenheimer, Kathleen Maguire-Zeiss

**Affiliations:** 1 Department of Neuroscience, Georgetown University School of Medicine, Washington, D.C., USA; 2 Jagiellonian Center of Innovation, Krakow, Poland

**Keywords:** Matrix metalloproteinase, MMP, Toll-like receptor, TLR, Tumor necrosis factor, TNF, MyD88, Microglia

## Abstract

**Background:**

Matrix metalloproteinases (MMPs) are a family of enzymes that are typically released from intracellular stores to act on specific extracellular substrates. MMP expression and activity can be increased in a neuronal activity-dependent manner, and further increased in response to tissue injury. MMP substrates include cell adhesion molecules (CAMs) that are abundantly expressed in the brain and well positioned for membrane proximal cleavage. Importantly, CAM integrity is important to synaptic structure and axon-myelin interactions, and shed ectodomains may themselves influence cellular function.

**Methods:**

In the present study, we have examined proteolysis of N-cadherin (N-cdh) by MMP-7, a family member that has been implicated in disorders including HIV dementia, multiple sclerosis, and major depression. With in vitro digest assays, we tested N-cdh cleavage by increasing concentrations of recombinant enzyme. We also tested MMP-7 for its potential to stimulate N-cdh shedding from cultured neural cells. Since select CAM ectodomains may interact with cell surface receptors that are expressed on microglial cells, we subsequently tested the N-cdh ectodomain for its ability to stimulate activation of this cell type as determined by nuclear translocation of NF-κB, Iba-1 expression, and TNF-α release.

**Results:**

We observed that soluble N-cdh increased Iba-1 levels in microglial lysates, and also increased microglial release of the cytokine TNF-α. Effects were associated with increased NF-κB immunoreactivity in microglial nuclei and diminished by an inhibitor of the toll-like receptor adaptor protein, MyD88.

**Conclusions:**

Together, these in vitro results suggest that soluble N-cdh may represent a novel effector of microglial activation, and that disorders with increased MMP levels may stimulate a cycle in which the products of excess proteolysis further exacerbate microglial-mediated tissue injury. Additional in vivo studies are warranted to address this issue.

## Background

Matrix metalloproteinase (MMP)-dependent proteolysis is increasingly recognized as an important effector of physiological plasticity, and learning and memory in particular [1]. However, since MMP activity can be dramatically upregulated with CNS injury, the potential for excessive enzyme activity to contribute to tissue injury must be considered. This latter possibility is supported by numerous in vivo studies in which broad-spectrum MMP inhibitors reduce brain injury in response to ischemia, viral infection, inflammatory stimuli, genetic disorders such as fragile X syndrome, and neurotoxicant exposure [2–4].

In terms of the mechanisms by which MMPs influence tissue injury, many studies have focused on their potential to influence blood brain barrier (BBB) permeability. Indeed, MMPs can cleave collagens that are critical components of the BBB [5–7]. MMPs can also influence BBB permeability in mouse models of ischemia and inflammation [8, 9]. It should be noted; however, that excess proteolysis of non-BBB targets might also contribute to MMP-dependent tissue injury.

Transmembrane cell adhesion molecules (CAMs) represent an important class of MMP substrates [1, 10]. CAMs are highly expressed on neurons and glial cells, and these molecules are well positioned to be targeted by MMPs following enzyme release from intracellular stores. CAM integrity may be critical to physiological synaptic plasticity, axon-myelin interactions, and reactive synaptogenesis [11–13]. Reduced CAM integrity has been linked to a reduction in synapse formation during the regenerative phase that follows brain injury [13]. In addition, and the focus of studies pursued herein, CAM fragments may also represent potent bioactive molecules [10, 14].

MMP-dependent CAM cleavage typically occurs proximal to the transmembrane domain, to release the major portion of the extracellular domain [15, 16]. The shed N-terminal fragment (NTF) is often relatively stable, in that elevated levels of shed NTFs can be detected in cerebrospinal spinal fluid samples from patients with CNS injury and/or inflammation [17, 18]. CAM fragments could thus represent a class of damage-associated molecular patterns (DAMPs) [19, 20].

Receptors for DAMPs include toll-like receptors (TLRs). These receptors are highly expressed on the microglia, resident innate immune cells of the brain, and TLRs are known to engage extracellular matrix protein fragments that have homology to CAM ectodomain regions. Moreover, the existence of cleavage-generated TLR ligands is not without precedent. For example, in *Drosophila*, extracellular proteolysis generates Spatzle, which then acts as a ligand for a TLR-analogous immune and pattern recognition receptor [20–22].

With respect to CNS inflammation and injury, TLR signaling has been shown to stimulate microglial release of neurotoxic molecules including the pro-inflammatory cytokine tumor necrosis factor-α (TNF-α). TLR agonists have also been linked to inflammasome activation [23, 24]. TLR expression may be increased in the inflamed CNS [25], and TLR signaling through MyD88 may be critical to expression of select disease states. It is therefore not surprising that innate immunity and TLR activation, in particular, are thought to play an important role in the murine model of multiple sclerosis, a prototypic inflammatory condition [25]. Cells of the innate immune system, including the microglia, stimulate myelin reactive T cells, and depletion of macrophages or microglia leads to a marked attenuation of experimental autoimmune encephalomyelitis (EAE) despite the adequate T cell sensitization [26, 27]. In addition, recent studies have linked microglial activation to cortical or hippocampal injury [28, 29]. Microglial activation is also observed in HIV dementia and is thought to contribute to disease progression [30].

In the present study, we investigate shedding of N-cdh, a CAM that is relatively abundant in the CNS, using an in vitro digest system and a cell culture model. In addition, using a variety of in vitro approaches and primary murine microglial cell cultures, we investigate soluble N-cdh for its potential to stimulate specific correlates of classical microglial activation.

## Methods

### Reagents

Recombinant mouse N-cdh was purchased from R & D Systems (Minneapolis, MN; 6626-NC) and reconstituted in sterile phosphate buffered saline (PBS) prior to use. This construct contains the soluble ectodomain (Met1-Ala724), as well as a linker (IEGRMDP) and Glu98-Lys330 of mouse IgG_2A_. Recombinant protein used was from a lot that was tested for endotoxin, which was measured at 0.000648 EU per microgram protein. Recombinant active MMP-2 and MMP-7 were purchased from EMD Biosciences, Inc. (Billerica, MA), as was the MMP inhibitor GM-6001. ADAM-10 was purchased from R & D Systems (Minneapolis, MN) and reconstituted in sterile phosphate buffered saline (PBS) prior to use. Aliquots were stored at −80 °C. The RGDS peptide was purchased from Tocris Bioscience (Bristol, UK; 91037-65-9) and reconstituted in sterile PBS. Normal mouse IgG was purchased from Santa Cruz Biotechnology, Inc (Dallas, Tx; sc-2025). Primary antibodies: to the C-terminal region of the p65 subunit of NF-κB were purchased from Abcam (anti-NF-κB p65 rabbit polyclonal (ab31481); Cambridge, MA); to the Iba-1 were purchased from Wako Chemical USA, Inc. (anti-Iba1 rabbit polyclonal (016-20001); Richmond, VA); and to the GAPDH were purchased from EMD Millipore (anti-GAPDH mouse monoclonal IgG_1_ (MAB374); Billerica, MA). For immunocytochemistry, Alexa Fluor® secondary antibodies were purchased from Molecular Probes/Thermo Fisher Scientific (Waltham MA). For western blot analyses, horseradish peroxidase-conjugated secondary antibodies were purchased from EMD Millipore (goat anti-mouse-HRP, AP130P) and EMD Millipore (goat anti-rabbit-HRP, AP132P). The nuclear counterstain, DAPI, was purchased from Thermo Fisher Scientific (62247). Lyophilized MyD88 inhibitor peptide and its control peptide were from Imgenex (San Diego, CA) and reconstituted in sterile PBS to a working concentration of 5 mM. Antibody to N-cdh was purchased from Millipore (Billerica, MA).

### In vitro digests

Unless otherwise noted, in vitro digests were performed as previously described using 2.5 μg recombinant N-cdh and 0.5 μg recombinant active enzyme [5]. Recombinant human MMP-2 and MMP-7 were purchased from EMD Millipore (catalog numbers PF023 and 444270, respectively; Billerica, MA), while recombinant human ADAM-10 was purchased from R & D Systems (936-AD; Minneapolis, MN). The MMP activity inhibitor GM-6001 was purchase from Tocris (catalog number 2983; Bristol, U.K.) and used at a final concentration of 2 μM. Reactions were terminated by addition of denaturing loading buffer and a 95 °C incubation (5 min) followed by freezing. Digestion products were resolved by electrophoresis on a 4–15% Tris-glycine polyacrylamide gradient gel and then transferred to a polyvinylidene difluoride (PVDF) membrane. The membrane was subsequently stained with Coomassie Blue.

### Cell culture and stimulation

#### B35 cells

The B35 rat neuroblastoma cell line [31] was obtained from the American Type Culture Collection (Manassas, Virginia), and cultures were maintained in DMEM supplemented with glucose, penicillin/streptomycin, and 10% fetal bovine serum at 5% CO_2_. Cultures were at 75–80% confluence at treatment. This cell line has been extensively characterized and used in previous studies related to neural CAMs (NCAM) [32]. These cells were treated with vehicle or 500 ng/ml recombinant MMP-7 for 1 or 6 h as indicated.

### Primary microglia

Mice were housed and treated in accordance with the guidelines of the Georgetown University Animal Care and Use Committee. Primary murine microglial cells were prepared from C57BL/6 P1-P3 pups as previously described [33] which results in a cell culture purity >95%. Briefly, mixed glial cultures were grown for 14–17 days from the cortices of neonatal murine pups (P1-3) in Microglial Complete Medium (Minimum Essential Medium Earle’s, supplemented with 1 mM l-glutamine, 1 mM sodium pyruvate, 0.6% *v*/*v* D-(+)-glucose, 100 μg/mL penicillin/streptomycin, 4% *v*/*v* fetal bovine serum, and 6% *v*/*v* horse serum). After 14–17 days in vitro, the microglia were isolated via rotary shaking (200 RPM; 37 °C; 5 h) and subsequently plated at a density of 1 × 10^5^ cells per well (24-well plates) in 0.5 mL of Microglial Growth Media (Minimum Essential Medium Earle’s, supplemented with 1 mM sodium pyruvate, 0.6% *v*/*v* D-(+)-glucose, 1 mM l-glutamine, 100 μg/mL penicillin/streptomycin, and 5% *v*/*v* fetal bovine serum) and subjected to experimentation as described below 24 h after plating.

Unless otherwise noted, microglial cell cultures were stimulated with 75 nM recombinant N-cdh in MGM. This concentration was effective in preliminary dose-response experiments in which the microglia were exposed to 15, 37.5, or 75 nM recombinant N-cdh, and TNF-α levels in the conditioned media were measured by ELISA. Using an ANOVA, differences between the control and stimulated groups became significant at 75 nM (control 14.75 ± 0.65 S.E.M., 15 nM: 25.75 ± 5.15 S.E.M., 37.5 nM: 103.2 ± 31.5, and 75 nM: 350.6 ± 130.8); therefore, 75 nM was chosen for the studies presented herein. For the TNF-α ELISA experiments, microglia were treated with PBS, 75 nM of N-cdh, heat-inactivated N-cdh (85 °C, 10 min), or normal mouse IgG (0.4 μg/well) for 24 h and the conditioned media retained and assayed as described below. Microglia used for the integrin blocking experiments were pretreated for 30 min in the presence or absence of 500 μM RGDS followed by exposure to N-cdh or PBS for 24 h. TNF-α release was quantified in the conditioned media.

#### Lysates and Western blot

Lysates from cultured cells were prepared via the addition of lysis buffer [50 mM Tris-HCl, pH 7.5, 150 mM NaCl, 0.1% sodium dodecyl sulfate (SDS), 1% NP-40, 0.5% sodium deoxycholate, 0.2 mM phenylmethylsulfonyl fluoride (PMSF), and 1× protease inhibitor cocktail (Sigma P8340)]. The mixture was placed into a microfuge tube, sonicated for 10 s, kept on ice for 20 min, and then spun at 14,000 rpm for 15 min at 4 °C in a microcentrifuge. Supernatants were then saved and used in Western blot experiments. Western blot was performed using 40 μg of protein per lane for B35 cell lysates or 12 μg per lane for primary microglial cell lysates. Prior to analysis, samples were mixed with sample buffer containing 5% β-mercaptoethanol and boiled for 5 min at 95 °C. Electrophoresis was performed on Tris-glycine polyacrylamide gradient gels (Biorad, Hercules, CA). Following electrophoretic transfer of the protein to nitrocellulose or PVDF (Iba-1 experiments), membranes were stained with Ponceau S to ensure equal loading and transfer. Membranes were then blocked in 5% nonfat dry milk in Tris-buffered saline with 0.1% Tween (TBST) for 1 h. The blot was then probed with the indicated primary antibody, at a dilution recommended by the manufacturer (see figure legend for specific dilutions), overnight at 4 °C. After washing the membrane three times (15 min each) in TBST, antigen-antibody complexes were incubated with the appropriate HRP-conjugated secondary antibody for 1–2 h at room temperature. The membrane was then washed again in TBST, followed by TBS and immunoreactive bands were visualized using electrochemiluminescence (Amersham/GE Healthcare Life Science; Piscataway, NJ).

### ELISA

TNF-α, pro-matrix metalloproteinase-9 (MMP-9), interleukin-6 (IL-6), and monocyte chemoattractant protein-1 (MCP-1) protein concentrations in cell culture supernatants were quantified using commercially available enzyme-linked immunosorbent assay (ELISA) kits according to the manufacturer’s instructions (R&D Systems; Minneapolis, MN).

#### Immunocytochemistry (NF-κB)

Microglia were plated on glass coverslips (1 × 10^5^ cells/coverslip; 12 mm; Deckglaser, Germany), stimulated as described, and subsequently processed for immunocytochemistry. More specifically, following treatment, cells were washed with PBS for 5 min, fixed with PBS containing 4% (*w*/*v*) paraformaldehyde and 4% (*w*/*v*) sucrose (pH 7.4) at room temperature for 15 min, permeabilized in PBS containing 0.1% (*v*/*v*) triton X-100 for 5 min, and blocked for 1 h with PBS containing 10% (*v*/*v*) goat serum. Cells were subsequently incubated overnight at 4 °C with rabbit anti-NF-κB (p65; 1:1000, Abcam; Cambridge, MA) in blocking buffer. This antibody was generated against amino acids 532-551 of the p65 subunit. Antibody/antigen complexes were visualized following incubation with Alexa Fluor® 594 conjugated goat anti-rabbit IgG secondary antibody (1:1000; Molecular Probes/Thermo Fisher Scientific; Waltham MA) in PBS containing 0.1% (*v*/*v*) triton X-100 and 1% goat serum. Unbound 2° antibody was removed by washing with PBS containing 0.1% (*v*/*v*) triton X-100. Cells were counterstained with 4′,6-diamidino-2-phenylindole (DAPI; 13.0 ng/μL; Thermo Fisher Scientific) in PBS for 5 min followed by two washes with PBS. Coverslips were mounted with Citifluor (Ted Pella, Redding, CA) and sealed with nail polish. Cells were imaged using a Zeiss Axioskop fluorescent microscope (Carl Zeiss; Thornwood, NY). For nuclear signal quantification, images of NF-κB and DAPI were captured from five distinct, randomly selected regions of each treatment condition for 25–60 cells per treatment group. Quantification of nuclear NF-κB intensity was completed as previously described [34, 35] utilizing Image J software (National Institutes of Health; Bethesda, MD).

#### MyD88 homodimerization inhibition

Primary microglia were plated as described above (1 × 10^5^ cells/coverslip). Cells were subsequently pre-treated with 200 μM of control peptide or MyD88 inhibitory peptide (Imgenex; San Diego, CA) for 30 min, followed by exposure to 75 nM recombinant N-cdh for 2 h. Cells were subsequently processed for immunocytochemistry as described above, and supernatants collected for ELISA.

#### Statistical analysis

Data were analyzed with Graphpad Prism software using a two-tailed *t* test or ANOVA for comparisons of more than two groups. Significance was determined as *p* < 0.05.

## Results

### Cleavage of N-cdh by MMP-7

**Fig. 1:**
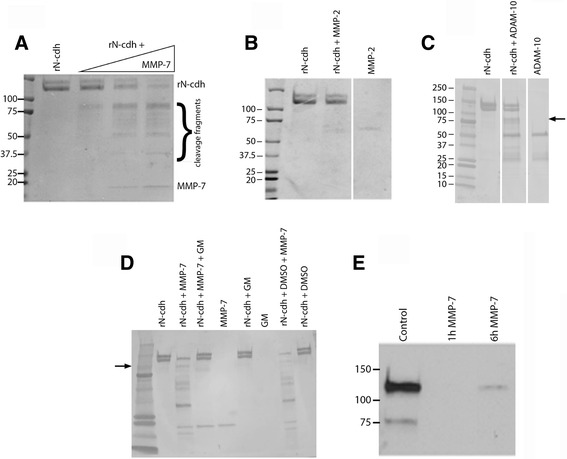
Cleavage of N-cdh by MMP-7. In (**a**) recombinant N-cdh (2.5 μg) was incubated with or without MMP-7 for 2 h at 37 °C. Digestion products were resolved by polyacrylamide gel electrophoresis, transferred to nitrocellulose, and then stained with Coomassie Blue (**a**). MMP-7 was used at increasing concentrations of 0.5, 1, and 1.5 μg/ml. In (**b** and **c**), a similar experiment was performed with MMP-2 or ADAM-10, respectively. While we do not observe appreciable cleavage with MMP-2 (**b**), as indicated by the *arrow* (**c**), ADAM-10 generated cleavage fragments of approximately 90 kDa. MMP-2 and ADAM-10 digests were run on the same gel for lanes shown in each figure but separated from other lanes for clarity. In (**d**), we demonstrate that the hydroxamate MMP inhibitor GM-6001 (GM) prevents MMP-mediated N-cdh cleavage, as expected, and that DMSO, in which GM is solubilized, does not impair generation of cleavage fragments including that observed at approximately 90 kDa (*arrow*). In (**e**), the integrity of N-cdh was also examined by Western blot using lysates from simultaneously prepared control and MMP-7-treated B35 cell culture wells. As indicated, time points at 1 and 6 h following treatment were examined in treated or control culture lysates. A reduction in full-length protein is observed within 1 h of MMP-7 treatment

In previous work, we have observed increased levels of MMP-7 in association with HIV dementia [36]. MMP-7 has also been linked to neuroinflammation with multiple sclerosis and depression [37–39]. Moreover, MMP-7 inhibitors have been shown to reduce N-cdh shedding from vascular smooth muscle cells [40]. Initial experiments were therefore performed to examine cleavage of recombinant N-cdh with increasing concentrations of MMP-7. Results are shown in Fig. 1a and suggest that MMP-7 generates discreet cleavage fragments. Though the identity of precise cleavage sites cannot be inferred without sequence analysis, the presence of discreet high molecular weight bands is consistent with proteolytic cleavage that generates long portions of the ectodomain. The band at approximately 90 kDa is consistent with the described propensity for MMPs to cleave CAMs at membrane proximal sites while smaller bands are consistent with additional cleavage sites within the full N terminal domain [1, 15]. Shown in Fig. 1b, c are results from in vitro digests using MMP-2 and ADAM-10, respectively. The latter is known to stimulate ectodomain shedding of N-cdh [41] and, similar to MMP-7, it generates cleavage fragments of approximately 90 kDa (arrow). In contrast, in our experimental system, MMP-2 did not generate cleavage fragments. Shown in Fig. 1d are results from digests performed using 1.25 μg rN-cdh and 0.5 μg rMMP-7 in the presence or absence of 2 μM GM-6001, an inhibitor of MMP activity. As can be appreciated, GM-6001 inhibited MMP-7-associated cleavage of recombinant N-cdh. An equivalent amount of DMSO (3.8%), in which GM-6001 was dissolved, did not inhibit MMP-7-mediated cleavage (rN-cdh + DMSO + MMP-7).

To investigate potential MMP-mediated cleavage of membrane-tethered N-cdh and shedding from neuronal cells in particular, subsequent studies tested MMP-7 for its ability to stimulate N-cdh shedding from a neuronal cell line [31]. We used 500 ng/ml MMP-7 as MMP levels in the nanogram per milliliter range have been detected in cerebrospinal fluid samples of patients with HIV dementia. As shown in Fig. 1e, treatment of B35 neuronal cells with MMP-7 was associated with a rapid reduction in detectable full length N-cdh immunoreactivity in cell lysates, consistent with release of ectodomain into the culture supernatant.

### Soluble N-cdh acts on primary microglial cells to stimulate nuclear translocation of NF-κB and increased levels of Iba-1 in lysates

Ectodomain shedding of CAMs would be expected to alter trans-cellular adhesive interactions [42]. In addition, a subset of CAM C-terminal fragments (CTFs) can influence gene transcription [16, 43]. A non-mutually exclusive mechanism by which CAM shedding could substantially influence cell biology involves the potential for soluble CAMs to interact with previously unengaged cell surface receptors to stimulate intracellular signaling cascades [10, 14, 44, 45].

**Fig. 2:**
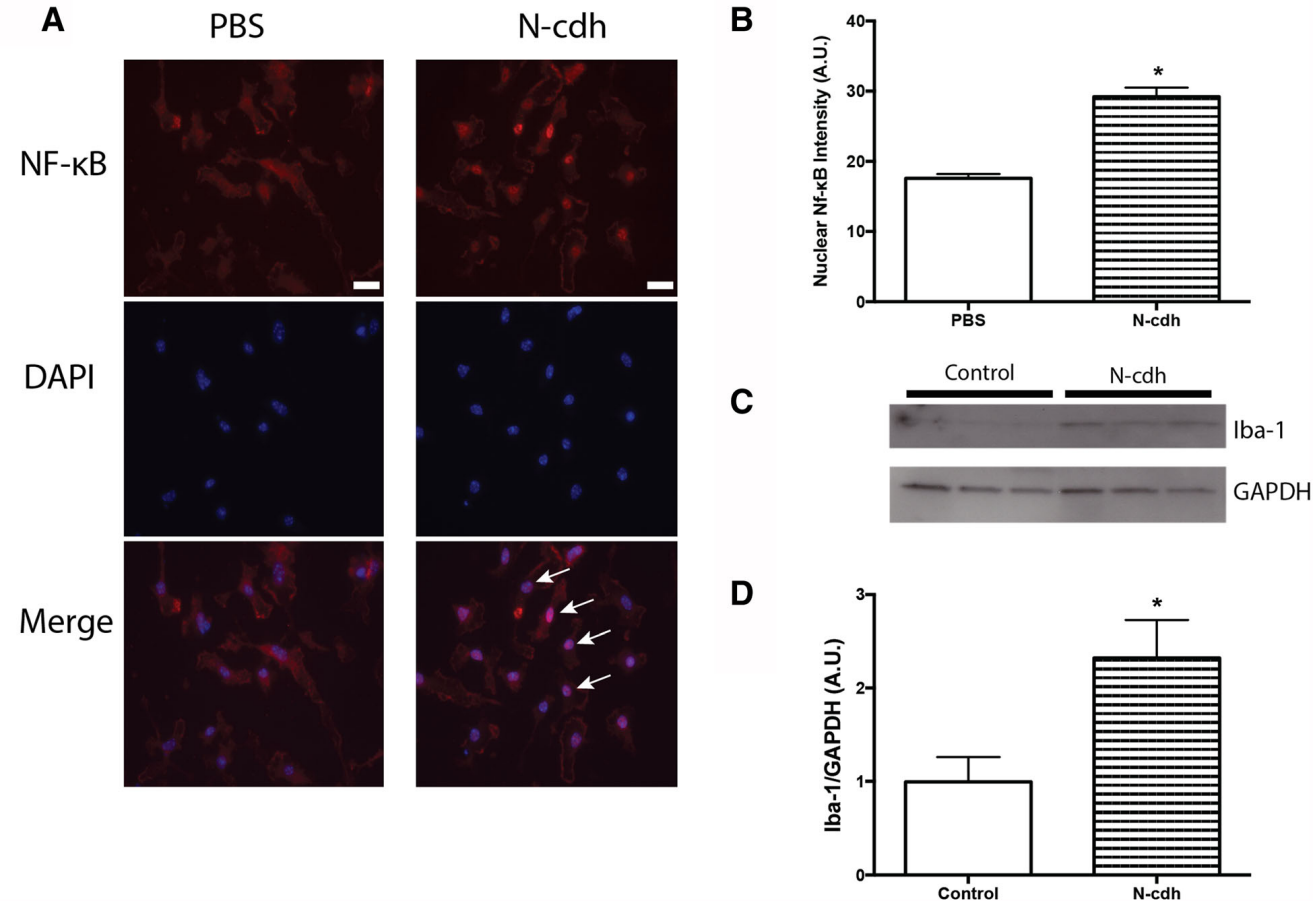
Soluble N-cdh acts on primary microglial cells to stimulate nuclear translocation of NF-κB and increased levels of Iba-1 in lysates. Microglia were immunofluorescently labeled for the p65 subunit of NF-κB (rabbit polyclonal anti-p65, 1:1000; Alexa Fluor 594-conjugated goat anti-rabbit IgG secondary antibody, 1:1000) and counterstained with DAPI, following a 2-h exposure to either vehicle (PBS) or 75 nM of soluble N-cdh. In comparison to control conditions, incubation of microglia with soluble N-cdh induced a robust increase in nuclear NF-κB p65 immunoreactivity, consistent with classical microglial activation. Representative images are shown in (**a**) and quantification in (**b**) (*p* < 0.01). The scale bar in (**a**) (*white*) represents 20 μm. Western blot analysis (**c**) shows that Iba-1 protein levels were also increased in microglial lysates (12 μg protein/lane; anti-Iba1 rabbit polyclonal, 1/1000; HRP-goat anti-rabbit IgG, 1/3000) following an 18-h exposure of microglia to 75 nM of N-cdh. Membranes were subsequently reprobed for GAPDH as a loading control (anti-GAPDH mouse monoclonal IgG_1_, 1/20,000; HRP-goat anti-mouse IgG, 1/2000). Data in (**d**) show results of densitometric analysis comparing changes in Iba-1 signal relative to GAPDH in control or N-cdh-stimulated cell lysates. The difference between the control and N-cdh-stimulated Iba-1/GAPDH density ratio is significant at *p* < 0.05. *Arrows* indicate increased nuclear NF-κB immunoreactivity

Microglial cells express relatively high levels of potential receptors for adhesion molecule fragments, including integrins and TLRs. Moreover, activation of this cell type has been increasingly implicated in the pathogenesis of neurodegenerative disorders including MS [29]. We therefore tested soluble N-cdh ectodomain for its ability to stimulate microglial activation as measured by increased nuclear translocation of NF-κB and expression of ionized calcium-binding adapter molecule, Iba-1, protein. As shown, soluble N-cdh stimulates a qualitative and quantitative increase in nuclear NF-κB immunoreactivity (Fig. 2a, b), as inferred by immunostaining for the p65 subunit and analysis of signal intensity. In addition, N-cdh stimulates an increase in Iba-1 levels in microglial lysates (Fig. 2c, d). As determined by densitometric analysis, the N-cdh stimulated increase in Iba-1 to GAPDH signal intensity is significant at *p* < 0.05.

### Soluble N-cdh increases release of microglial proteins including TNF-α

**Fig. 3:**
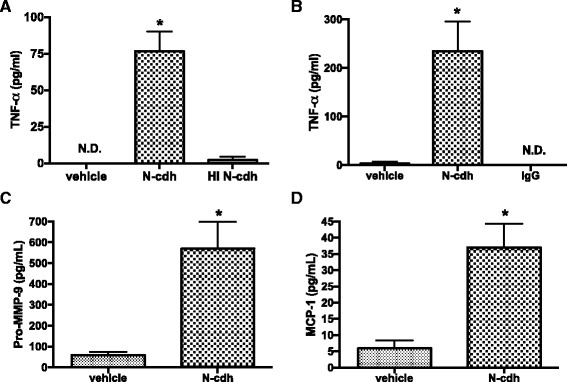
Soluble N-cdh increases microglial cell release of immune modulators including TNF-α. Supernatants from control or N-cdh-stimulated (75 nM, 18 h) microglia were tested by ELISA for TNF-α. Results from an experiment performed in triplicate with vehicle, N-cdh, or an equivalent amount of heat-inactivated protein (HI N-cdh; 95 °C for 30 min) are shown in (**a**) while results from a quadruplicate experiment with vehicle and IgG controls are shown in (**b**). The difference between control and N-cdh is significant at *p < 0.05 for both experiments. The difference between control groups is not significant for either. N.D. = not detectable. In (**c** and **d**), we show results from a similar experiment performed in triplicate with vehicle or N-cdh (75 nM, 18 h). In this experiment, supernatant levels of pro-MMP-9 or MCP-1 were evaluated as indicated, and a significant increase in both was observed

Though microglial cells are heterogeneous in terms of their phenotype, recent studies suggest that populations may be relatively polarized with “classically activated” populations described as M1 cells and alternatively or de-activated populations described as M2 [46]. Increased release of the pro-inflammatory cytokine TNF-α can follow from NF-κB signaling and is typically associated with M1 polarization, which in turn may be particularly inimical in the setting of neurodegeneration and neuroinflammation [47]. As shown in Fig. 3a, b, release of TNF-α is significantly increased in N-cdh-stimulated microglia. In contrast, equivalent concentrations of heat-inactivated N-cdh or IgG control or PBS treatment did not have a significant effect on TNF-α release (Fig. 3a, b). Due to limitations associated with increased release of a single soluble activation marker, additional experiments were performed to assess N-cdh-dependent release of MMP-9 and MCP-1. Results are shown in Fig. 3c, d and demonstrate that N-cdh can more generally enhance release of potential inflammatory mediators.

### Pre-treatment of microglial cells with an integrin antagonist does not abrogate N-cdh-stimulated TNF-α release

Similar to their full-length counterparts, shed CAMs can interact with cell surface receptors through homophilic and heterophilic interactions. In previous studies, we and others have observed that shed CAMs can bind to unengaged integrins to in turn stimulate changes in intracellular signaling [14, 44, 48].

**Fig. 4:**
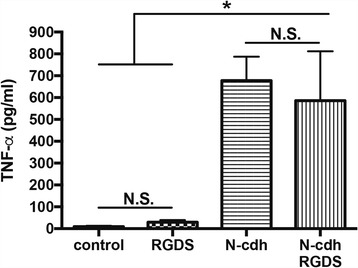
Pre-treatment of microglial cells with an integrin antagonist does not abrogate N-cdh-stimulated TNF-α release. An RGDS peptide was tested for its potential to attenuate N-cdh-stimulated TNF-α release from primary microglial cells. Supernatants from vehicle control (PBS), RGDS pre-treated (500 μM, 30 min pretreatment), and N-cdh (75 nM) with or without RGDS pre-treatment (500 μM, 30 min pretreatment) were collected 18 h later and analyzed by ELISA. The mean and standard error are shown in Fig. 4, and difference between pre-treated and non pre-treated N-cdh groups was not significant (N.S.). Supernatant from both groups that received N-cdh had significantly higher levels of TNF-α compared to control and RGDS alone (*n* = 4 per group; *p < 0.05)

Microglia express a variety of integrins including α_5_β_1_, an integrin that has been linked to both microglial activation and increased expression of the pro-inflammatory molecule MMP-9 [49]. This is one of several integrins that recognizes ligands with an RGD amino acid sequence [50]. We therefore tested an RGDS peptide, which blocks the activation of RGD-binding integrins, for its potential to abrogate N-cdh-stimulated TNF-α release. The microglia were pretreated for 30 min in the presence or absence of 500 μM RGDS followed by exposure to N-cdh or PBS for 24 h. TNF-α release was quantified in the conditioned media. Results, shown in Fig. 4, demonstrate that this peptide does not abrogate N-cdh-stimulated TNF-α release in our experimental system.

### A peptide inhibitor of MyD88-dependent TLR signaling, abrogates N-cdh-stimulated NF-κB translocation and TNF-α release

**Fig. 5:**
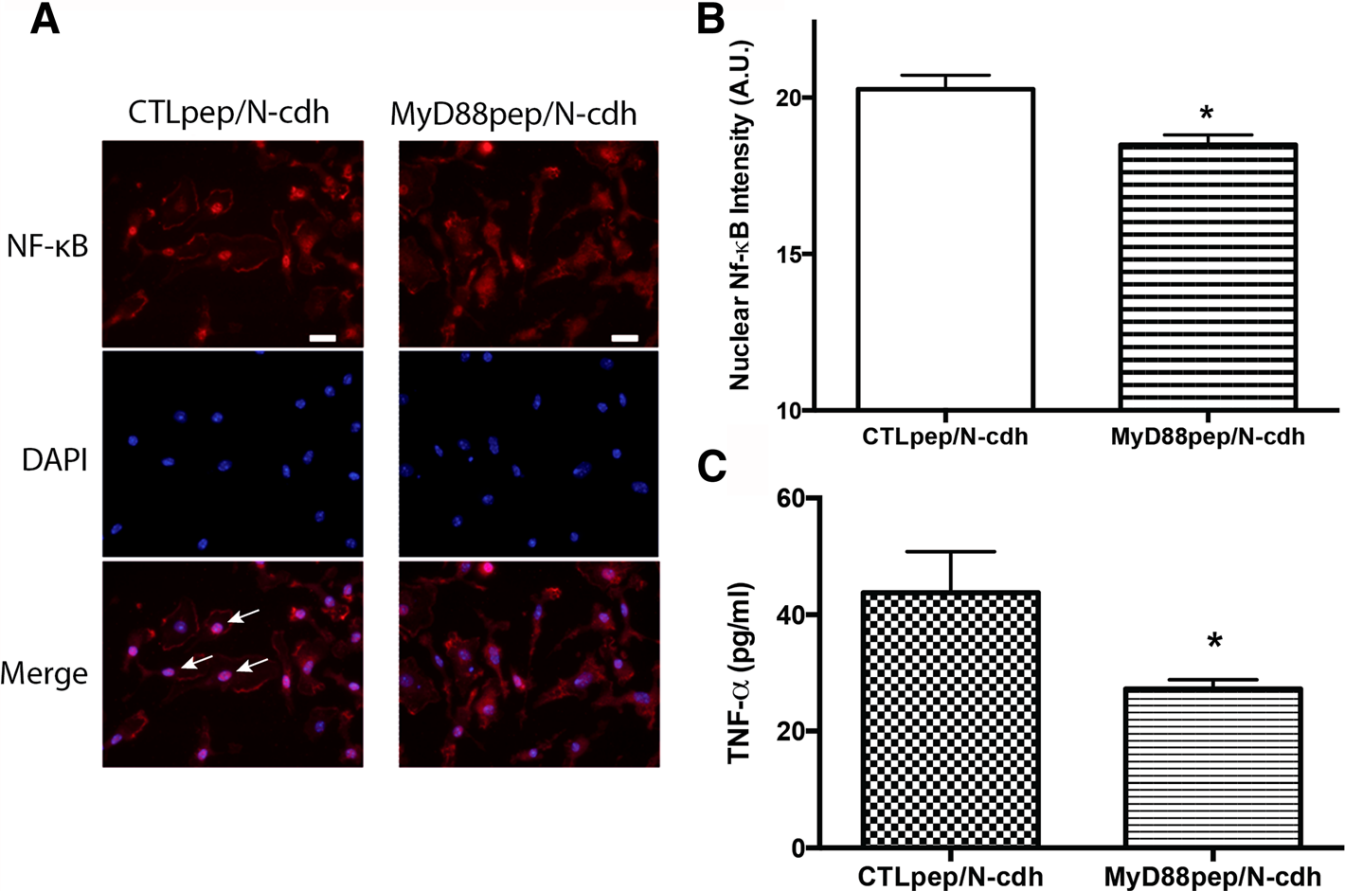
Inhibition of MyD88-dependent TLR signaling abrogates N-cdh-stimulated TNF-α release and NF-κB translocation. Microglia were pre-treated with either 200 μM of control peptide (CTLpep) or inhibitory MyD88 peptide (MyD88pep) for 30 min, followed by a 2-h exposure to 75 nM soluble N-cdh (**a**–**c**). Supernatants were collected and fixed cells were immunostained for NF-κB (rabbit polyclonal anti-p65, 1:1000; Alexa Fluor 594-conjugated goat anti-rabbit IgG secondary antibody, 1:1000) and counterstained with DAPI, while cell culture supernatants were subsequently analyzed for TNF-α protein concentration via ELISA (**c**). Data shown is representative and from one of two separate experiments each performed in triplicate. As shown, MyD88 inhibition led to a reduction in N-cdh-stimulated NF-κB translocation. Representative immunostaining is shown in (**a**) and quantification of nuclear signal intensity change is shown in (**b**) (*n* = 60 control and 84 MyD88 inhibitor-treated cells). The difference between nuclear signal intensity in vehicle and N-cdh is significant at *p < 0.05. The *scale bar* again represents 20 μm. In addition, as shown in (**c**), inhibition of MyD88 homodimerization resulted in a significant decrease in N-cdh-stimulated TNF-α release from microglia in comparison to control conditions (*p < 0.05). *Arrows* in CTLpep indicate nuclear NF-κB, which is diminished with the inhibitory MyD88 peptide

TLRs represent a class of pattern recognition receptors (PRRs) that are highly expressed on microglia and linked to classical activation. PRRs are activated by varied exogenous ligands called pathogen-associated molecular patterns (PAMPs) which include triacyl lipopeptides, flagellin, dsDNA, ssRNA, and bacterial lipopolysaccharides [51]. More recently, these same TLRs have been shown to bind endogenous ligands, referred to as damage-associated molecular patterns (DAMPs), molecules that are typically exposed or increased with tissue injury [19, 20, 25, 52]. Since soluble CAM levels are increased with tissue injury [53–55], and have regions homologous to matrix fragments that have been implicated in TLR signaling [20], CAM fragments could represent a class of DAMPs. We therefore examined a peptide inhibitor of MyD88 homodimerization, an event necessary for signaling through a subset of TLRs, for its potential to abrogate N-cdh-stimulated NF-κB translocation and TNF-α release. Because NF-κB translocation occurs relatively quickly, in these experiments, we examined changes in both NF-κB and TNF-α at 2 h, a timepoint that we previously demonstrated to be appropriate for both p65 nuclear translocation and TNF-α release [56]. As shown in Fig. 5, inhibition of MyD88 homodimerization reduced N-cdh-stimulated increases in both endpoint measures. These data suggest that TLR signaling may contribute to N-cdh-mediated microglial activation.

## Discussion

MMPs represent a family of enzymes initially named for their potential to remodel extracellular matrix [57, 58]. Expression and/or release of select MMPs is increased in the background of neuroinflammation [59]. Published studies have demonstrated that MMP activity can cleave BBB tight junction and basement membrane components to facilitate the CNS ingress of specific leukocyte subpopulations [7]. Recently published work posits that MMPs can produce DAMPs through their potential to process large ECM proteins such as hyaluron [60, 61]. Less well studied is the question of whether increased MMP activity contributes to disease pathogenesis through excessive CAM cleavage.

CAMs represent an important target for cell-associated and cell-secreted MMPs. In vitro and in vivo studies suggest that metalloproteinase activity is concentrated at the cell surface [62, 63] and emerging evidence links CAM shedding to important physiological and pathological endpoints [48, 64]. CAM integrity is important to synaptic stability, axon-myelin interactions, and reactive synaptogenesis following injury [13, 42]. In addition, shedding of specific CAMs is linked to the generation of intracellular domains that influence gene transcription [43]. For example, N-cdh shedding leads to the generation of a CTF that inhibits CREB-dependent gene transcription [16]. A third and non-mutually exclusive consequence of CAM shedding, and one that is increasingly appreciated in recent studies, is the possibility that shed NTFs are themselves potent effectors of diverse normal and pathological processes. These fragments are relatively stable, as suggested by studies in which varied CAM NTFs can be detected in cerebrospinal fluid samples from normal and/or diseased individuals [53–55, 65]. Similar to their full-length counterparts, shed CAMs can likely interact with cell surface receptors through homophilic and heterophilic interactions [14, 66]. This is supported by published work in which soluble ICAM-5 can interact with neuronal surface molecules including full-length ICAM-5 and β_1_ integrins [14, 15].

TLRs represent another class of cell surface molecules that might be engaged by soluble CAMs as these PRRs are activated by injury-associated molecular patterns (DAMPs) [19, 20, 25, 52]. Soluble CAM levels may be increased with brain injury [53–55], and these molecules have regions homologous to matrix components that have been implicated in TLR signaling [20]. CAM fragments could therefore represent a class of DAMPs.

In the present study, we show that MMP-7 can cleave recombinant N-cdh. Similar to ADAM-10, which has been demonstrated to stimulate ectodomain shedding of N-cdh [41, 67, 68], MMP-7 generates a fragment of approximately 90 kDa. Though sequencing studies would be confirmatory, given that recombinant N-cdh contains the entire ectodomain and is of similar molecular weight to native protein, this result is consistent with the described ability of MMPs to cleave CAMs at a membrane proximal position. Ectodomain shedding is further supported by loss of N-terminal N-cdh immunoreactivity in lysates from MMP-7-treated neural cells. While our focus was on MMP-7, due to overlapping substrate specificity, we acknowledge that additional MMPs might stimulate N-cdh shedding in select settings. A subset of family members is expressed in the brain and, like MMP-7, may be upregulated with inflammation [69].

Herein, we also show that soluble N-cdh can stimulate microglial activation as demonstrated by varied endpoints including increased expression of Iba1, nuclear localization of NF-κB, and release of TNF-α. Moreover, we suggest that TLR signaling in particular can contribute to N-cdh-stimulated microglial activation, since endpoint measures are significantly reduced by an inhibitor of MyD88 signaling. Though inhibition is incomplete, it should be noted that blocking MyD88 signaling with peptides or siRNA typically has partial effects in terms of attenuating TLR signaling [52, 70, 71]. Changes in overall TNF-α levels are of interest, as is increased nuclear localization of a transcription factor linked to inducible TNF-α expression. Importantly, however, while both likely reflect microglial activation, the two are not necessarily causally related. Transcription factors additional to NF-κB are important to TNF-α. Moreover, TNF-α can be released from preformed stores [72]. Of additional note, N-cdh also increased expression and/or release of additional proteins that may be regulated in an NF-κB-dependent manner: MCP-1 and MMP-9 [73, 74].

Soluble N-cdh-dependent microglial activation is of interest. Classical microglial activation has been implicated as a mediator of hippocampal and synaptic injury in varied disease models. A recent study, which focused on hippocampal dysfunction in a mouse model of multiple sclerosis, noted that a loss of inhibitory interneurons occurred with associated microglial activation and a relative lack of infiltrating blood-borne immune cells [29]. Recent work has also shown that MMP-7 is increased with major depression [75], a condition that is increasingly associated with microglial activation [76]. That N-cdh stimulated an increase in TNF-α release is of interest in that this cytokine is typically associated with classical or pro-inflammatory M1 polarization. Though M1 as opposed to immunoregulatory or anti-inflammatory M2 polarity is a complex distinction, and characteristics of each are not mutually exclusive, it is worth noting that the M1 phenotype may be inimical to neuronal repair [47].

The potential for an MMP-generated fragment to activate microglia also provides a potential mechanistic link for studies that have shown reduced microglial activation in the background of MMP inhibition. For example, tetracycline and tetracycline derivatives can directly inhibit the activity of already formed MMPs via chelation of the active site zinc atom [77]. Tetracycline derivatives are now used in varied disease models to reduce microglial activation. It is thus tempting to speculate that reduced microglial activation follows, at least in part, from reduced levels of microglial-activating CAM fragments.

Herein, we also show that specific cell surface receptors may be important to soluble N-cdh-dependent microglial activation. As compared to other resident cells of the CNS, microglia express high levels of two receptor classes that have been linked to their activation, integrins and TLRs [78, 79]. Our studies suggest a role for the latter in N-cdh-dependent effects. We cannot, however, rule out an effect for other cell surface molecules including CAM-binding fibroblast growth factor (FGF) receptors [80]. In addition, though an antagonist for RGD-binding integrins did not block N-cdh-stimulated TNF-α release in our system, it would be premature to exclude a role for integrins in MMP-dependent microglial activation. For example, N-cdh could interact with non-RGD-binding integrins that are linked to microglial activation. Moreover, damage-associated MMPs might stimulate shedding of additional CAMs, such as ICAM-5, whose NTFs might engage RGD and non-RGD-binding microglial integrins [78, 81].

While pathological shedding of CAMs may stimulate widespread and injurious microglial activation, it is important to consider that regulated shedding might be important to physiological microglial functions. For example, since MMPs can be released in a neuronal activity dependent manner, it is tempting to speculate that regulated CAM-dependent microglial activation could potentially contribute to physiological synaptic pruning [82].

## Conclusions

In summary, our data suggest that N-cdh shedding can be stimulated by MMP-7 and that soluble N-cdh represents a novel effector of classical microglial activation. Future in vivo studies to better understand the consequences of disease-associated CAM shedding are warranted.

## Abbreviations

CAM: Cell adhesion molecule
CTF: C-terminal fragment
DAMP: Damage-associated molecular pattern
ELISA: Enzyme-linked immunosorbent assay
FGF: Fibroblast growth factor
Iba-1: Ionized calcium binding adaptor molecule-1
ICAM: Intercellular adhesion molecule
MMP: Matrix metalloproteinase
N-cdh: N-cadherin
NTF: N-terminal fragment
PVDF: Polyvinylidene difluoride
TLR: Toll-like receptor
TNF-α: Tumor necrosis factor-α
